# Future Directions Concerning the Impact of Childhood and Adolescent Adversities in the Field of Men’s Mental Health: The New York Declaration

**DOI:** 10.3389/fpubh.2015.00004

**Published:** 2015-01-19

**Authors:** Timothy R. Rice, Zoltan Rihmer, Julia Golier, Leo Sher

**Affiliations:** ^1^Icahn School of Medicine at Mount Sinai, New York, NY, USA; ^2^National Institute of Psychiatry and Addictions, Semmelweis University, Budapest, Hungary; ^3^James J. Peters Veterans’ Administration Medical Center, Bronx, NY, USA

**Keywords:** men’s mental health, testosterone, child development, traumatology, psychotherapy

In November 2014, the New York-based Members of the World Federation of Societies of Biological Psychiatry’s Task Force on Men’s Mental Health, Timothy Rice M.D., Julia Golier M.D., Leo Sher, M.D., and Zoltan Rihmer, M.D., Ph.D. of Budapest, Hungary developed a statement on future directions concerning the impact of childhood and adolescent adversities in the field of men’s mental health. We wished to describe the unique vulnerability of boys and young men to childhood physical abuse and other types of psychological trauma and to develop therapeutic recommendations to address this vulnerability. The following assessments regarding the current state of knowledge in the field were made.

## Targets of Physical Abuse and Trauma

Whereas girls are most likely to be the victims of sexual abuse, boys are much more likely to experience physical abuse and trauma (1). This stands across all levels of abuse severity, from slapping and hitting to kicking in, burning, and scalding (2). Boys are much more likely to be admitted to hospitals for serious physical abuse than girls (*P* < 0.0001) (3). Male youth are additionally more likely to witness and to be involved in urban violence than females (4), with low-income children and adolescents are at greatest risk (5).

## Central Nervous System Vulnerability

Abuse directly alters the physiology of the developing central nervous system (6) and disrupts development across multiple functional domains, including the physiological, cognitive, emotional, and social (7). For example, abuse confers specific deficits within the emotion regulation system (8), a neural system that is fundamentally implicated in childhood psychiatric disorders (9). Because testosterone and other male sex hormones also disrupt this system through both direct organizational and activational effects upon its neural correlates (10), the consequences of abuse may have a lower threshold to clinically present in boys relative to girls. Male sex hormones additionally effect global delays in central nervous system structural maturation and development (11). These delays present to boys a greater window of developmental vulnerability to internal and environmental insult relative to girls. Vulnerable components include dopaminergic tracts and other central nervous system subsystems, which may account for the increased prevalence of attention-deficit hyperactivity disorder (ADHD) among boys than among girls (11). We may deduce that this increased vulnerability to insult includes an increased vulnerability to the environmental insults of abuse and trauma.

Select populations of men may be at even greater vulnerability. For example, the short allele of the serotonin transporter gene (5-HTTLPR), which produces lower/dysregulated central serotonin turnover, is significantly associated with cyclothymic and irritable affective temperaments (12). Individuals who carry the short allele and who have experienced childhood trauma are more vulnerable not only for later major depression but also for impulsive, hostile, irritable, and aggressive personality traits (13, 14). The constellation of trauma and the 5-HTTLPR is a well-documented precursor of auto- and hetero-aggressive behavior (14, 15).

Suicide attempters with cyclothymic and irritable affective temperaments report significantly more frequently childhood physical and/or sexual abuse (16). Major mood disorder patients who report childhood physical or sexual abuse show higher rate of irritable and cyclothymic affective temperaments and higher Beck hopelessness score than those without this adversity (17). These findings suggest that besides impulsivity (18), cyclothymic or irritable temperaments are further mediating variables between these early negative life events and adult suicidal behavior. This gene–environment interplay may be of specific importance to men given their increased rates of physical abuse and trauma relative to women.

## Neuroendocrinological Vulnerability

Trauma in children additionally affects the hypothalamic–pituitary–adrenal (HPA) axis by increasing basal cortisol levels and reducing HPA responsivity to stressors (19). Preliminary evidence suggests that boys may be more vulnerable to these trauma-induced alternations of normal physiology than girls (20, 21). These alterations have negative effects on both the body (cushinoid response, immunological dysfunction) and the mind. For example, chronically elevated corticosteroid concentrations impair serotonergic neurotransmission and raise risk for psychopathology (22). Boys’ vulnerability to abuse may be best understood within a unified mind–brain medical model (23).

## Externalizing Behavior

Perhaps, as a consequence of these neurophysiologic considerations, following trauma, girls present to their environment as more adjusted (24). Girls experience more internalizing symptoms (depression, anxiety, and post-traumatic stress disorder symptoms) than their male counterparts, and thus they generally receive less environmental attention. The increased attention which externalizing symptoms and behaviors grab toward boys is regrettably often negative. Externalizing behaviors can induce anger in parents and contribute to the differential aggression directed at boys relative to girls (25). When parental anger manifests as abuse, a positive-feedback cycle is produced that invites further externalization and further abuse (26).

Too often, externalizing children may be labeled “bad” by parents, health care workers, and legal professionals alike. They may be placed in traditional behavioral management programs, which reduce target behaviors (27) but which may not optimally address the developmental deficits wrought by childhood trauma, including those in emotion regulation (8). These individuals may not appreciate the role of trauma and emotion regulation deficits in these children; they may additionally be unaware of or have no access to the major evidence-based treatments for childhood trauma (Table 1). In many cases, children with externalizing behaviors are placed on antipsychotics (28), which may have significant physiological and psychological consequences for these children while failing to address the underlying effects of abuse and trauma. Children are more vulnerable to the metabolic and endocrinologic adverse effects of these agents relative to adults (29, 30). Boys may be particularly affected by the psychological consequences of these medications, which include long-term meanings concerning autonomy and motivation. Boys with histories of abuse and trauma have a special need to maintain a sense of who they are, of their rights to autonomy and privacy, of their humanity, and of the meaningfulness of their experiences (31). Insufficient attention to traumatic etiologies underlying externalizing behaviors may be particularly developmentally deleterious in the boys and young men who go on to become men of this world.

**Table 1:** **Interventions for childhood trauma rated as either effective or promising (32)**.

Child–Parent Psychotherapy (CPP)	Dyadic, attachment-based intervention for children up to age 5 traditionally employed over year-long course in weekly sessions. Developed from infant–parent psychotherapy within psychoanalytic perspective
Trauma Affect Regulation: Guide For Education and Therapy (TARGET)	Seven-step skill-based intervention over 10–12 individual or group sessions. Targets trauma-induced responses employing the FREEDOM acronym (Focus, Recognize triggers, Emotion self-check, Evaluate thoughts, Define goals, Options, and Make a contribution)
Trauma-Focused Cognitive-Behavior Therapy (TF-CBT):	12–16 session intervention employing the PRACTICE acronym (Psychoeducation and parenting skills, Relaxation, Affective expression and regulation, Cognitive coping, Trauma narrative development and processing, *In vivo* gradual exposure, Conjoint parent–child sessions, and Enhancing safety and future development) through child, parent, and conjoint sessions.
Cognitive-Behavioral Intervention for Trauma in Schools (CBITS)	Skill-based intervention with interpersonal elements for application in middle and high school settings in 10 group sessions
Parent–Child Interaction Therapy (PCIT)	Combines behavioral, play therapy, and parent education for children aged 2–7 years and their caregivers. Two stages (relationship development and discipline training) enable benefits of traditional play therapy and behavioral treatment.
Trauma and Grief Component Therapy for Adolescents (TGC T-A)	10–24 sessions for adolescents aged 12–20 years drawing from draws upon cognitive-behavioral theory and social provisions theory.

## The Justice System

The juvenile justice system is overwhelmingly a male-populated system (33). These youth have a much higher frequency of trauma histories throughout the world, including sub-Saharan Africa (34). A history of trauma places children at a much greater risk for placement in the juvenile justice system (33). In America, as many as 95% of youth in the juvenile justice system had experienced trauma, and there is a direct correlation between traumatic exposure and the degree of delinquency (35) mostly among this male population.

Among male adolescents in juvenile detention placements, a history of abuse was significantly correlated with having fathered a child as an adolescent (36). Addressing trauma may prevent perpetuation of a cycle of insufficient resources for children. These adolescent fathers may have special needs and be a particular target for helpful interventions (37). Preexisting services for adolescent fathers (38) may be a unique means of helping both adolescent males and vulnerable young male infants and toddlers who carry the gendered vulnerabilities to trauma that we have discussed.

Given these findings and assessments, the following recommendations were made.

### Preventions to reduce child/adolescent adversities

Education is paramount. Both the lay public and professionals involved in the wellbeing of children will benefit from education concerning the unique vulnerability of male children and adolescents to physical abuse and trauma. Public health initiatives are of benefit to the lay public. Fathers should be integrated into population-based interventions (39), despite the difficulties with which such an aim is appreciated to entail (40). Providers in all of the diverse service systems involved in the wellbeing of children and youth, including legal professionals, child welfare agencies, the foster system, and beyond, should have sufficient training. They should understand the role of abuse and trauma in childhood as it pertains to men’s overall development. Police and mental healthcare professions should receive training in promoting fluid, cooperative interactions in helping abused children (41). Healthcare worker and physician interventions for trainees and the experienced alike (42) are of help in producing improvements in achievement, attitudinal change, and clinical behavior (43). Trainees may be a particularly good target for these interventions, with well-defined areas for improvement among emergency medicine, family medicine, and pediatric residents (44), among others. Review literature for these populations (45) is additionally of help. The special role that abuse and trauma may play in the mental health of boys and young men should be emphasized; in particular the role that externalizing behaviors in young boys may play in relation to trauma should be emphasized as a central point of men’s mental health among children and young men.

### Interventions to reduce negative results of childhood adversities

In addition to education, it is essential to develop interventions tailored to the full spectrum of male youth, including male infants, toddlers, children, adolescents, and young men. Fortunately, there are already multiple of diverse theoretical backgrounds and perspectives to help children (Table 1). Treatments, which focus on the specific needs of male youth, need to be developed both through flexibly tailoring preexisting treatments to the special needs of this population and through the development of novel therapeutics that are sensitive to the mental health needs of male children and youth.

Male children with a history of trauma may be disruptive, externalizing, and difficult to engage in traditional therapy. Male adolescents are less likely to seek any health care (46). When they do, those with traditionally masculine self-identities view traditional psychotherapy as less acceptable than executive coaching (47). These facts concerning male youth should inform the interventions to children and adolescents in order to make them more accessible and more accommodating to the disruptive male child or the avoidant adolescent. Therapies can integrate flexibility to accommodate the disruptive behaviors of children, and provide mental health professionals with a means of addressing the externalizing behaviors of children. Clinicians must be encouraged to recognize the negative feelings which the disruptive child can invoke and to use these feelings productively and prevent him or herself from enactment of the abused parent–child dyadic relationship, which for the abused child may be the only means available of relating. For male adolescents, therapies should be constructed and promulgated in accordance with positive masculine norms and which directly address avoidance behaviors and anxieties. Emphasizing the development of health and virility within a psychotherapeutic program as well as targeting misconceptions among males that a lifestyle involving poor self-care is masculine may be of value. Continued integration of these efforts via multidisciplinary treatment planning and cross-discipline education into populations with male youth with a high likelihood of having histories of trauma (foster care, juvenile justice system) will significantly increase accessibility.

Novel therapeutics should be built upon the increasing knowledge of men’s mental health in children and adolescents. For example, the finding that boys with a history of trauma may be particularly vulnerable to environmentally induced deficits in the emotion regulation system (8) suggests that interventions targeting this neural system may be of particular use. Regulation-Focused Psychotherapy for Children with Externalizing Behaviors [RFP-C; (27)] is one such novel manualized 12- to 16-session intervention developed to be used with this population. The integration of traditional methods of child psychotherapy with a brain-based model of dimensional neuropsychiatric pathology that is compatible with the Research Domain Criteria project [RDoC; (28, 29)] of the National Institute of Mental Health (NIMH) permits a targeted intervention for this children that is capable of hypothesis testing within a classification system based on observable behaviors with neural correlates. As the organization and activational effects of male sex hormones on the male central nervous system and on the male emotion regulation system become increasingly understood, there will be increased value of organizing a psychotherapeutic approach, which works in tandem with biological neuropsychiatric models of psychopathology.

Prescribers, who employ medication in the amelioration of suffering of young male children and adolescents, should have an understanding of the psychological and physical adverse effects of these agents. They should carefully weigh these risks against the benefits of these agents prior to initiating treatment. The inability of these agents to directly address the traumatic etiology of many disruptive behaviors should be kept close to consciousness, and interventions, which do address childhood abuse and trauma should be used concurrently. These include both psychotherapeutic and social support services as well as alternate psychopharmacotherapeutics, such as the selective serotonin reuptake inhibitors (SSRIs) and other serotonergic agents. An increasing understanding of how these agents address brain-based neuropsychiatric deficits in this population will lead to increasing synchrony between mind-based (e.g., psychotherapeutic) and brain-based (e.g., pharmacotherapeutic) interventions.

This declaration serves as a preliminary communication of select members of the World Federation of Biological Psychiatry’s Task Force on Men’s Mental Health concerning future directions for research, clinical care, and public health policy concerning the importance of men’s mental health in boys and young men.

## Conflict of Interest Statement

The authors declare that the research was conducted in the absence of any commercial or financial relationships that could be construed as a potential conflict of interest.

## References

[1] Tolin DF Foa EB. Sex differences in trauma and posttraumatic stress disorder: a quantitative review of 25 years of research. Psychol Bull (2006) 132:959–92. 10.1037/0033-2909.132.6.959 17073529

[2] Molnar BE Buka SL Brennan RT Holton JK Earls F. A multilevel study of neighborhoods and parent-to-child physical aggression: results from the project on human development in Chicago neighborhoods. Child Maltreat (2003) 8:84–97. 10.1177/1077559502250822 12735711

[3] Leventhal JM Martin KD Gaither JR. Using US data to estimate the incidence of serious physical abuse in children. Pediatrics (2012) 129:458–64. 10.1542/peds.2011-1277 22311999

[4] Zona K Milan S. Gender differences in the longitudinal impact of exposure to violence on mental health in urban youth. J Youth Adolesc (2011) 40:1674–90. 10.1007/s10964-011-9649-3 21400207

[5] Wade R Shea JA Rubin D Wood J. Adverse childhood experiences of low-income urban youth. Pediatrics (2014) 134:e13–20. 10.1542/peds.2013-2475 24935995

[6] Glaser D The effects of child maltreatment on the developing brain. Med Leg J (2014) 82:97–111 10.1177/0025817214540395 25228749

[7] Toth SL Cicchetti D A developmental psychopathology perspective on child maltreatment. Introduction. Child Maltreat (2013) 18:135–9 10.1177/1077559513500380 23886641 PMC4520222

[8] Kim J Cicchetti D. Longitudinal pathways linking child maltreatment, emotion regulation, peer relations, and psychopathology. J Child Psychol Psychiatry (2010) 51:706–16. 10.1111/j.1469-7610.2009.02202.x 20050965 PMC3397665

[9] Rice TR Hoffman L. Defense mechanisms and implicit emotion regulation: a comparison of a psychodynamic construct with one from contemporary neuroscience. J Am Psychoanal Assoc (2014) 62:693–708. 10.1177/0003065114546746 25082875

[10] Rice TR Sher L Testosterone, emotion regulation and childhood aggression. Neuropsychiatry (2013) 3(3):267–70 10.2217/npy.13.26

[11] Martel MM Klump K Nigg JT Breedlove SM Sisk CL. Potential hormonal mechanisms of attention-deficit/hyperactivity disorder and major depressive disorder: a new perspective. Horm Behav (2009) 55:465–79. 10.1016/j.yhbeh.2009.02.004 19265696 PMC3616481

[12] Gonda X Rihmer Z Zsombok T Bagdy G Akiskal KK Akiskal HS. The 5HTTLPR polymorphism of the serotonin transporter gene is associated with affective temperaments as measured by TEMPS-A. J Affect Disord (2006) 91:125–31. 10.1016/j.jad.2005.12.048 16464506

[13] Vergne DE Nemeroff CB. The interaction of serotonin transporter gene polymorphisms and early adverse life events on vulnerability for major depression. Curr Psychiatry Rep (2006) 8:452–7. 10.1007/s11920-006-0050-y 17094925

[14] Gonda X Fountoulakis KN Csukly G Bagdy G Pap D Molnár E Interaction of 5-HTTLPR genotype and unipolar major depression in the emergence of aggressive/hostile traits. J Affect Disord (2011) 132:432–7. 10.1016/j.jad.2011.03.029 21492940

[15] Sarchiapone M Jaussent I Roy A Carli V Guillaume S Jollant F Childhood trauma as a correlative factor of suicidal behavior – via aggression traits. Similar results in an Italian and in a French sample. Eur Psychiatry (2009) 24:57–62. 10.1016/j.eurpsy.2008.07.005 18774698

[16] Rihmer A Szilágyi S Rózsa S Gonda X Faludi G Rihmer Z The role of childhood abuse in adult suicidal behaviour. Neuropsychopharmacol Hung (2009) 11(4):237–46. 20150661

[17] Pompili M Iliceto P Innamorati M Rihmer Z Lester D Akiskal HS Suicide risk and personality traits in physically and/or sexually abused acute psychiatric inpatients: a preliminary study. Psychol Rep (2009) 105:554–68. 10.2466/PR0.105.2.554-568 19928616

[18] Braquehais MD Oquendo MA Baca-García E Sher L. Is impulsivity a link between childhood abuse and suicide? Compr Psychiatry (2010) 51:121–9. 10.1016/j.comppsych.2009.05.003 20152291

[19] Tarullo AR Gunnar MR. Child maltreatment and the developing HPA axis. Horm Behav (2006) 50:632–9. 10.1016/j.yhbeh.2006.06.010 16876168

[20] Vigil JM Geary DC Granger DA Flinn MV. Sex differences in salivary cortisol, alpha-amylase, and psychological functioning following Hurricane Katrina. Child Dev (2010) 81:1228–40. 10.1111/j.1467-8624.2010.01464.x 20636692

[21] Trickett PK Gordis E Peckins MK Susman EJ. Stress reactivity in maltreated and comparison male and female young adolescents. Child Maltreat (2014) 19:27–37. 10.1177/1077559513520466 24482544

[22] Leonard BE. HPA and immune axes in stress: involvement of the serotonergic system. Neuroimmunomodulation (2006) 13:268–76. 10.1159/000104854 17709948

[23] Renoir T Hasebe K Gray L. Mind and body: how the health of the body impacts on neuropsychiatry. Front Pharmacol (2013) 4:158. 10.3389/fphar.2013.00158 24385966 PMC3866391

[24] Villodas MT Litrownik AJ Thompson R Jones D Roesch SC Hussey JM Developmental transitions in presentations of externalizing problems among boys and girls at risk for child maltreatment. Dev Psychopathol (2014):1–15. 10.1017/S0954579414000728 25045912 PMC4302043

[25] Jouriles EN Norwood WD Physical aggression toward boys and girls in families characterized by the battering of women. J Fam Psychol (1995) 9:69–78 10.1037/0893-3200.9.1.69

[26] Patterson GR The aggressive child: victim and architect of a coercive system. In: Hamerlynck L Handy L Mash E, editors. Behavior Modification and Families: Theory and Research. New York, NY: Brunner/Mazel (1976) p. 267–316.

[27] Kazdin AE Rotella C The Kazdin Method for Parenting the Defiant Child: With No Pills, No Therapy, No Contest of Wills. Boston, MA: Houghton Mifflin (2008).

[28] Olfson M Blanco C Liu S-M Wang S Correll CU. National trends in the office-based treatment of children, adolescents, and adults with antipsychotics. Arch Gen Psychiatry (2012) 71:81–90. 10.1001/archgenpsychiatry.2012.647 22868273

[29] Correll CU Metabolic side effects of second-generation antipsychotics in children and adolescents: a different story? J Clin Psychiatry (2005) 66(10):1331–2 10.4088/JCP.v66n1018 16259549

[30] Maayan L Correll CU. Weight gain and metabolic risks associated with antipsychotic medications in children and adolescents. J Child Adolesc Psychopharmacol (2011) 21:517–35. 10.1089/cap.2011.0015 22166172

[31] Cohen DJ Tourette’s syndrome: a model disorder for integrating psychoanalysis and biological perspectives. Int Rev Psychoanal (1991) 18:195–208.

[32] Pilnik L Kendall J Victimization and trauma experienced by children and youth: implications for legal advocates. Moving from Evidence to Action: The Safe Start Series on Children Exposed to Violence, Issue Brief #7. North Bethesda, MD: (2012).

[33] Espinosa EM Sorensen JR Lopez MA. Youth pathways to placement: the influence of gender, mental health need and trauma on confinement in the juvenile justice system. J Youth Adolesc (2013) 42:1824–36. 10.1007/s10964-013-9981-x 23824982

[34] Atilola O Omigbodun O Bella-Awusah T. Lifetime exposure to traumatic events among adolescents in contact with the Nigerian juvenile justice systems compared with a comparison group of secondary school students. Paediatr Int Child Health (2014) 34:92–100. 10.1179/2046905513Y.0000000113 24621244

[35] Becker SP Kerig PK. Posttraumatic stress symptoms are associated with the frequency and severity of delinquency among detained boys. J Clin Child Adolesc Psychol (2011) 40:765–71. 10.1080/15374416.2011.597091 21916694

[36] Khurana A Gavazzi S. Juvenile delinquency and adolescent fatherhood. Int J Offender Ther Comp Criminol (2011) 55:756–70. 10.1177/0306624X10372109 20508087

[37] Gordon DM Watkins ND Walling SM Wilhelm S Rayford BS. Adolescent fathers involved with child protection: social workers speak. Child Welfare (2011) 90:95–114. 22533056

[38] Curran L. Social work and fathers: child support and fathering programs. Soc Work (2003) 48:219–27. 10.1093/sw/48.2.219 12718417

[39] Osborn M Working with fathers to safeguard children: failure to work with fathers around the child occurs regularly. Child Abuse Negl (2014) 38:993–1001 10.1016/j.chiabu.2014.05.004 24931758

[40] Taylor J Daniel B. The rhetoric vs. the reality in child care and protection: ideology and practice in working with fathers. J Adv Nurs (2000) 31:12–9. 10.1046/j.1365-2648.2000.01265.x 10632788

[41] Marans S Berkowitz SJ Cohen DJ. Police and mental health professionals. Collaborative responses to the impact of violence on children and families. Child Adolesc Psychiatr Clin N Am (1998) 7:635–51. 9894059

[42] Menoch M Zimmerman S Garcia-Filion P Bulloch B. Child abuse education: an objective evaluation of resident and attending physician knowledge. Pediatr Emerg Care (2011) 27:937–40. 10.1097/PEC.0b013e3182307ae5 21960095

[43] Forbes EE Christopher May J Siegle GJ Ladouceur CD Ryan ND Carter CS Reward-related decision-making in pediatric major depressive disorder: an fMRI study. J Child Psychol Psychiatry (2006) 47:1031–40. 10.1111/j.1469-7610.2006.01673.x 17073982 PMC2129133

[44] Starling SP Heisler KW Paulson JF Youmans E. Child abuse training and knowledge: a national survey of emergency medicine, family medicine, and pediatric residents and program directors. Pediatrics (2009) 123:e595–602. 10.1542/peds.2008-2938 19273504

[45] Kodner C Wetherton A Diagnosis and management of physical abuse in children. Am Fam Physician (2013) 88:669–75. 24364482

[46] Marcell AV Ford CA Pleck JH Sonenstein FL. Masculine beliefs, parental communication, and male adolescents’ health care use. Pediatrics (2007) 119:e966–75. 10.1542/peds.2006-1683 17403834 PMC2488152

[47] McKelley RA Rochlen AB Conformity to masculine norms and preferences for therapy or executive coaching. Psychol Men Masc (2010) 11:1–14 10.1037/a0017224

[48] Hoffman L Rice T Regulation-Focused Psychotherapy for Children with Externalizing Behaviors (RFP-C). New York, NY: Routledge (2015).

[49] Insel TR The NIMH research domain criteria (RDoC) project: precision medicine for psychiatry. Am J Psychiatry (2014) 171:395–7 10.1176/appi.ajp.2014.14020138 24687194

[50] Insel T Cuthbert B Garvey M Heinssen R Pine DS Quinn K Research domain criteria (RDoC): toward a new classification framework for research on mental disorders. Am J Psychiatry (2010) 167:748–51 10.1176/appi.ajp.2010.09091379 20595427

